# Association of Retinal Layer Thickness with Brain Volume and Cortical Thickness in older adults

**DOI:** 10.1002/alz.091127

**Published:** 2025-01-09

**Authors:** Ji Won Han, Hyeong Min Kim, Min Jeong Kwon, Jieun Park, Jun Sung Kim, Se Joon Woo, Ki Woong Kim

**Affiliations:** ^1^ Seoul National University Bundang Hospital, Seongnam Korea, Republic of (South); ^2^ Seoul National University College of Medicine, Seoul Korea, Republic of (South); ^3^ Seoul National University College of Medicine, Seoul National University Bundang Hospital, Seongnam Korea, Republic of (South); ^4^ Seoul National University College of Natural Sciences, Seoul Korea, Republic of (South)

## Abstract

**Background:**

To elucidate the biological mechanisms underpinning the association between macular Retinal Nerve Fiber Layer (RNFL) thickness and cognitive function in older adults, this study investigates its correlation with brain volume and cortical thickness.

**Method:**

From a community‐dwelling prospective cohort, we included 166 non‐demented participants aged over 65 years (mean age 75.2 ± 5.3; 61.4% women) who had 3D T1 MRI images and no ocular pathologies that could affect retinal layer thickness. Using spectral‐domain optical coherence tomography, we assessed the thickness of the macular RNFL. We analyzed the relationship between RNFL thickness and regional brain volumes using voxel‐based morphometry, adjusting for age, sex, and total intracranial volume (uncorrected p < 0.001, cluster size threshold = 100 voxels). The relationship between RNFL thickness and cortical thickness by FreeSurfer was assessed using Pearson's correlation analysis, with adjustments for age and sex.

**Result:**

Increased gray matter volumes correlating with outer RNFL thickness were present in the right inferior parietal (t=3.81), the left superior frontal (t=3.71), and left inferior temporal (t=3.95) cortices. The total RNFL thickness was associated with the volume of the right inferior parietal cortex (t=3.62). Significant associations were found between RNFL thickness and the cortical thickness of multiple brain regions. In particular, the cortical thickness in the left posterior cingulate and both supramarginal regions was significantly correlated with total, inner, and outer RNFL thickness. Upon adjusting for multiple comparisons, these associations did not retain statistical significance.

**Conclusion:**

RNFL thickness was associated with various brain regions involved in sensory information processing and integration, as well as cognitive functions. We also observed correlations with brain regions implicated in the early stages of Alzheimer's disease pathology.

